# Two in One: Use of Divalent Manganese Ions as Both Cross-Linking and MRI Contrast Agent for Intrathecal Injection of Hydrogel-Embedded Stem Cells

**DOI:** 10.3390/pharmaceutics13071076

**Published:** 2021-07-13

**Authors:** Lukasz Kalkowski, Dominika Golubczyk, Joanna Kwiatkowska, Piotr Holak, Kamila Milewska, Miroslaw Janowski, Joaquim Miguel Oliveira, Piotr Walczak, Izabela Malysz-Cymborska

**Affiliations:** 1 Department of Neurosurgery, School of Medicine, Collegium Medicum, University of Warmia and Mazury, 10-082 Olsztyn, Poland; lukasz.kalkowski@uwm.edu.pl (L.K.); dominika.golubczyk@uwm.edu.pl (D.G.); joanna.kwiatkowska@transpharmation.co.uk (J.K.); kamila.milewska@uwm.edu.pl (K.M.); 2 Department of Surgery and Radiology, Faculty of Veterinary Medicine, University of Warmia and Mazury, 10-719 Olsztyn, Poland; piotr.holak@uwm.edu.pl; 3 Center for Advanced Imaging Research, Department of Diagnostic Radiology and Nuclear Medicine, University of Maryland School of Medicine, Baltimore, MD 21201, USA; miroslaw.janowski@som.umaryland.edu (M.J.); pwalczak@som.umaryland.edu (P.W.); 4 a3B’s Research Group, I3Bs—Research Institute on Biomaterials, Biodegradables and Biomimetics, University of Minho, Headquarters of the European Institute of Excellence on Tissue Engineering and Regenerative Medicine, Avepark, Parque de Ciência e Tecnologia, Zona Industrial da Gandra, 4805-017 Guimarães, Portugal; miguel.oliveira@i3bs.uminho.pt; 5 ICVS/3B’s—PT Government Associate Laboratory, 4806-909 Guimarães, Portugal

**Keywords:** hydrogel, manganese, contrast agent, biomaterial, intrathecal, large animals

## Abstract

Cell therapy is a promising tool for treating central nervous system (CNS) disorders; though, the translational efforts are plagued by ineffective delivery methods. Due to the large contact surface with CNS and relatively easy access, the intrathecal route of administration is attractive in extensive or global diseases such as stroke or amyotrophic lateral sclerosis (ALS). However, the precision and efficacy of this approach are still a challenge. Hydrogels were introduced to minimize cell sedimentation and improve cell viability. At the same time, contrast agents were integrated to allow image-guided injection. Here, we report using manganese ions (Mn^2+^) as a dual agent for cross-linking alginate-based hydrogels and magnetic resonance imaging (MRI). We performed in vitro studies to test the Mn^2+^ alginate hydrogel formulations for biocompatibility, injectability, MRI signal retention time, and effect on cell viability. The selected formulation was injected intrathecally into pigs under MRI control. The biocompatibility test showed a lack of immune response, and cells suspended in the hydrogel showed greater viability than monolayer culture. Moreover, Mn^2+^-labeled hydrogel produced a strong T1 MRI signal, which enabled MRI-guided procedure. We confirmed the utility of Mn^2+^ alginate hydrogel as a carrier for cells in large animals and a contrast agent at the same time.

## 1. Introduction

Despite the continuous effort and growing number of newly developed drugs, diseases of the central nervous system (CNS) are particularly refractory to treatment, and many diseases are still incurable. Amyotrophic lateral sclerosis (ALS) is an excellent example of a deadly, fatal disease that affects the brain and spinal cord, leading to degeneration and death of upper and lower motor neurons. Replacement of lost motor neurons via transplantation is difficult, if not impossible, so more realistic therapeutic approaches are based on providing trophic support for neurons. One example of that strategy is the transplantation of glial progenitor cells [1,2]. Presently, ongoing clinical trials with stem cell transplantation in ALS patients are sporadic (a total of six registered in clinicaltrials.gov), with only four registered trials (NCT04651855, NCT03296501, NCT03268603, NCT03214146) that include local (intraspinal/intrathecal) cell delivery. Even though, none of them have the cells of neuronal lineage. Indeed, cell administration route is an essential and highly underappreciated issue. Among available gateways facilitating the local accumulation of cells in the brain is intra-arterial, stereotaxic, or intrathecal administration. All these techniques are feasible, but they are not without drawbacks. In intraarterial stem cell delivery, engraftment of naïve neural stem cells is relatively low and requires cell engineering [3,4]. In larger-sized mesenchymal stem cells, excellent engraftment has been shown with naïve cells in both small [5] and large animals [6]. The direct stereotaxic injection may be feasible for localized pathology, such as in Parkinson’s disease. Still, it is invasive, particularly in large lesions with an example of stroke or disseminated diseases such as ALS and MS. Multiple injections would be required, which amplify invasiveness and risk of complications. The intrathecal route is characterized by minimal invasiveness and an excellent safety profile with convenient access via lumbar puncture. Another advantage is that CSF space offers a large surface area of CNS for stem cells to migrate into parenchyma or act through the release of trophic factors. The feasibility, reproducibility, and safety of intrathecal stem cell delivery have been proven in small animals [7], large animals [8], and clinical settings [9]. However, the challenge with intrathecal injection is that the cells infused as a suspension are subject to sedimentation and may drain away from the injection site. Additionally, lack of adequate cell contact and trophic support hurt cell survival. Indeed, the vast majority of reported intrathecal cell injection studies are based on injecting cell suspension [9]. Consequently, there is a need to support the cells with a biomaterial matrix that mimics the natural environment of the tissue [10]. Biomaterial–cell composites could then be used to improve the precision and reproducibility of injection, allowing placement at the desired area of the spinal cord while also promoting cell survival and integration. In addition to being injectable, these biomaterials must be biocompatible and biodegradable. There are many classes of biomaterials available that can be used to support intrathecal cell transplantation.

As demonstrated by our team’s previous research, hyaluronan-based hydrogels can be supplemented with iron oxide-based MRI contrast agents facilitating image-guided delivery thus improving precision of deployment of tissue composites with real-time MRI, as shown in study performed in large animal models [11]. While iron-based contrast agents provide strong MRI signal, their disadvantages include interference with diagnostic imaging and hypointense signal that is difficult to interpret in longitudinal imaging due to similarity to the blood, air, or other artifacts. Contrast agents that result in the positive (hyperintense) signal are more desirable and in the context of biomaterials imaging, manganese ions in combination with alginate provide unique opportunities. Manganese, due to its effect on shortening T1 relaxation, leads to enhancement of the signal on T1 weighted scans. Another attractive feature of manganese (II) ions is their cross-linking ability triggering polymerization of the alginate. Alginates as bioscaffolds have been used to support transplanted cells [12]. They are biodegradable, of low immunogenicity, and are injectable [13,14,15,16]. Moreover, they can be supplemented with agents promoting cell survival and increasing the regenerative capacity [10].

In the present study, we focused on developing an alginate composition optimal for intrathecal stem cell transplantation, with supplementation of manganese (II) to facilitate non-invasive monitoring of the biomaterial by MRI. This approach will enable, for the first time, the protection of the therapeutic agents from the recipient’s immune system, and against sedimentation or displacement from the site of administration. Thanks to the dual functionality of the manganese ions, biomaterial acquires desirable biomechanical properties and MR visibility during transplantation, which will increase the safety and predictability of the procedure, and thus potentially improve the effectiveness of therapy.

## 2. Materials and Methods

### 2.1. Preparation of the Alginate Hydrogels and In Vitro MRI

Initially, we focused on solvent selection. For this purpose, the low viscosity sodium alginate (PRONOVA UP LVM; DuPont Nutrition Norge, NovaMatrix, Norway) was dissolved in 4.6% mannitol (diluted from 25% solution; Hospira, Lake Forest, IL, USA) or 0.9% NaCl to a 2% aqueous solution. The mixtures were then magnetically stirred until completely dissolved at room temperature. Then, the manganese chloride (Sigma Aldrich, Hamburg, Germany) solution was incorporated into alginate solutions to get the final concentrations: 0.05, 0.1, and 1 mM of MnCl_2_. Next, the alginate–manganese solutions were filtered over 0.22 μm filters (Greiner Bio-One, Rainbach, Austria). Subsequently, cross-linking agents, 0.5% solutions of calcium alginate beads (CaM, >75 μm; DuPont Nutrition Norge, NovaMatrix, Sandvika, Norway) were prepared in 4.6% mannitol and 0.9% NaCl and sterilized in the autoclave. Next, sodium alginate–manganese solutions of two diluents (mannitol and NaCl) were incorporated into separate syringes and connected with the syringes filled with cross-linkers (CaM beads dissolved in mannitol and CaM beads dissolved in NaCl, respectively) using a three-way stopcock. Hydrogels were cross-linked by mixing alginate and cross-linker; next, they were injected into the 15 mL Falcon tubes filled with artificial CSF (aCSF-), consisting of 119 mM NaCl, 26.2 mM NaHCO_3_, 2.5 mM KCl, 1 mM NaH_2_PO_4_, 1.3 mM MgCl_2_,10 mM glucose and 2.5 mM CaCl_2_. The aCSF without hydrogel was used as a control. Hydrogel phantoms were scanned using clinical 3 T scanner (Magnetom Trio, Siemens AG, Munich, Germany) in the standard head coil. Imaging intervals included one hour after preparation and then daily for up to 14 days. For T1-weighted images, TE was 10 ms, TR was 1111.4 ms, slice thickness was 2 mm with 2 mm interslice. The field of view was 15 × 15 cm. For T2-wieghted images, TE was 78 ms, TR was 5860 ms, with the same geometry as in T1. The image resolution was 0.46875 × 0.46875 µm for both T1 and T2 images. We used turbo-spin echo sequence with flip angles of 120° (T1) and 150°.

### 2.2. Immune Response to Hydrogels

To determine the immunoreactivity of the components of alginate hydrogels, the cell lines expressing toll-like receptors (TLR) were used: THP1-XBlue™-MD2-CD14, which expresses all Toll-like receptors (TLR) [17] and THP1-XBlue ™ -defMyD, does not respond through TLR2, TLR4, TLR5, TLR8, and IL-1Rs (InvivoGen, Toulouse, France). Cells were cultured in Roswell Park Memorial Institute 1640 medium (RPMI; Life Technologies, Warsaw, Poland), supplemented with 2 mM·l-glutamine, 1.5 g/L sodium bicarbonate, 4.5 g/L, 10 mM 4-(2-hydroxyethyl)-1-piperazineethanesulfonic acid (HEPES; Thermo Scientific, Warsaw, Poland), 1 mM sodium pyruvate (Thermo Scientific, USA), 10% heat-inactivated fetal bovine serum (FBS; Thermo Scientific, USA), 100 μg/mL Normocin™ (InvivoGen) and Penicillin/Streptomycin (50 μg/mL; Thermo Scientific, USA). Cells were plated at a concentration of 1 × 10^5^ cells/well on 96-wells plates (Thermo Scientific, USA) and co-incubated with samples of alginates and Ca beads of various sizes (DuPont Nutrition Norge, Sandvika, Norway; diluted in 4.6% mannitol) and cultured overnight at 37 °C and 5% CO_2_. The lipopolysaccharide from *Escherichia coli* (LPS-EK 0.4, 4, and 40 μg/mL, InvivoGen) was used as a positive control for the THP1-XBlue™-MD2-CD14 cell line and l-Ala-γ-D-Glu-mDAP (Tri-DAP 2, 8, 20 and 80 μg/mL, InvivoGen) for the THP1-XBlue™–defMyD88 cell line. The 4.6% mannitol added to RPMI 1640 was used as a negative control. The SEAP was quantified using QUANTI-Blue™ (InvivoGen) according to the manufacturer’s instructions.

### 2.3. In Vitro Characterization of the Viability of the Glial Restricted Progenitors (GRPs) Embedded in the LVM Hydrogels

To assess the survival of GRPs after embedding in LVM hydrogels luciferase-expressing mouse (msGRPs) were used. The msGRPs were isolated from light-producing transgenic mice as described previously [11]. Cells were embedded in the concentration of 1 × 10^5^ cells/50 µL LVM (diluted in 4.6% mannitol). After cross-linking with 50 µL of CaM (>75 µm; diluted in 4.6% mannitol), cell–hydrogel composites were plated on 96-well plates. After 10 min when the hydrogel was fully solidified, the GRP culture medium (as described previously [11]) was added to the well. As a control, monolayer cell culture without hydrogel was used. The relative luminescence was measured daily, with the addition of D-luciferin (Gold Biotechnology, St, Louis, MO, USA) to the medium (15 µg/mL) and reading luminescent signal using Infinite^®^ 200 plate reader (Tecan, Männedorf, Switzerland).

To analyze the influence of the addition of Manganese chloride into the hydrogels on msGRPs viability, luciferase-expressing mouse glial restricted progenitors (msGRPs; 1 × 10^5^ cells/well) were embedded in the 50 µL 2% LVM (4.6% mannitol) supplemented with 1 mM MnCl_2_ and cross-linked with 50 µL CaM (>75 µm; diluted in 4.6% mannitol). Next, hydrogels with cells were loaded onto 96-well plates and, after 10 min, covered with GRP culture media. Additionally, msGRPs ^Luc+^ were embedded in the 2% LVM without MnCl_2_ addition. As a control, monolayer cell culture without hydrogel was used with the addition of the MnCl_2_ (1 mM) and without. The relative luminescence was measured daily, every seven days, with the addition of D-luciferin as described above.

### 2.4. In Vitro Characterization of the Dependence of the MRI Signal on the Type of Hydrogel

Various hydrogels were tested to investigate their compositions on MRI signal. Based on previous experiments, three hydrogels (dissolved in 4.6% mannitol), that were able to pass through the MR compatible intrathecal catheter, were selected: 1.5% LVM/MnCl_2_ + 0.5% CaM, 2% LVM/MnCl_2_ + 0.5 CaM, 2% LVM/MnCl_2_ + 0.1% CaCl_2_. Hydrogels were injected through the catheter into the 50 mL Falcon tubes fulfilled with aCSF and scanned in the MR every day for 7 days. The MRI protocol included T1 (TR/TE 650/9.3 ms). As a control, the aCSF was scanned without the hydrogel injected.

### 2.5. Experimental Animals

All animal experiments were approved by the Local Ethics Committee in Olsztyn (56/2018 of 31 July 2018) and were performed according to ARRIVE guidelines. Eight juvenile, Large White domestic pigs (40 kg, both genders) were used. At least two weeks before transplantation, animals were acclimated to the new environment and human presence to minimize the stress associated with the experiment. Pigs had access to water and food ad libitum.

### 2.6. MRI-Guided Intrathecal Transplantation of LVM/Mn^2+^ Hydrogels

During the transplantation, pigs were anesthetized with a combination of sevoflurane (2%) and propofol (3 mg/kg/h). Animals were placed under C-arm and using X-ray guidance, MR-compatible catheter (16 G, Portex, Smiths Medical, Dublin, OH, USA) was navigated into a thoracic section of the spinal cord via lumbar puncture. Next, animals were transported into MRI and positioned supine in a 3 T MRI scanner (Magnetom Trio, Siemens AG, Germany). For anatomical images, we used T1- and T2-weighted turbo spin echo sequences. For T1-weighted images, a sequence with 650 ms TR and 9.3 ms TE, 0.83 × 0.83 µm resolution, 32 × 32 cm field of view and flip angle of 147° was used. For T2-weighted images, a sequence with 5360 ms TR and 117 ms TE, 0.83 × 0.83 µm resolution, 32 × 32 cm field of view and flip angle of 150° was used. Additionally, an isotropic T2-weighted SPACE sequence was used; with 1200 ms TR and 222 ms TE, 0.96 × 0.96 µm resolution, 37 × 37 cm field of view and 85° flip angle. The LVM/Mn^2+^ (2% in mannitol; 1 mM MnCl_2_) and CaM beads (0.5% in mannitol; >75 um) solutions were loaded into separate syringes. Immediately before transplantation, two solutions were mixed (total volume 4 mL) and injected manually.

During the hydrogel injection, a dynamic 2D T1-weighted FLASH sequence was used, a sequence with 23 ms TR and 2.7 ms TE, 1.5625 × 1.5625 µm resolution, 30 × 30 cm field of view and 48° flip angle. Images were acquired to monitor the biodistribution of hydrogel in real-time. The control T1- and T2-weighted MRI scans were acquired 24 h after transplantation to evaluate hydrogel visibility.

### 2.7. Histopathological Analysis

Tissues of pigs spinal cords were collected immediately after slaughter (next day post hydrogel transplantation), fixed in 4% paraformaldehyde, for 48 h at 4 °C, embedded in 30% sucrose, frozen on dry ice for 5 mins and kept at −80 °C until analyses. Next, tissues were sectioned at 20 μm using Hyrax C25 PLMC cryostat (Zeiss, Warsaw, Poland), transferred onto Superfrost slides (Menzel Gläser, Braunschweig, Germany), and processed for immunofluorescence. After drying at room temperature (RT) for 10 min, sections werewashed 2 times in phosphate-buffered saline (PBS) for 10 min and then blocked in a PBS with 10% NGS (Normal Goat Serum; Sigma Aldrich) for 1 h at RT. Next, slides were incubated overnight at 4 °C with primary rabbit polyclonal antibodies diluted in PBS/10% NGS (rabbit anti-CD4 1:50, rabbit anti CD45 1:50; rabbit anti CD68 1:50). The next day, slides were washed 3 times in PBS for 10 min, incubated with secondary antibody conjugated with fluorochrome Alexa 488 (Alexa Fluor 488 goat anti-rabbit IgG, 1:400; Thermo Fisher Scientific, Waltham, MA, USA) for 1.5 h at RT and washed in PBS. A negative control staining for the secondary antibody was done by replacing the primary antibodies with 10% NGS (Sigma Aldrich). Microscopic sections were covered with DAPI mounting medium (Fluoroshield, Sigma Aldrich) and analyzed using a Zeiss Axio Observer microscope (Zeiss, Germany).

### 2.8. Statistical Analysis

Statistical analyses were performed using GraphPad Prism 9.0 (GraphPad Software 2021, Inc., San Diego, CA, USA). The distribution of normality was evaluated with the Shapiro–Wilk test. The one-way ANOVA followed by Dunnett’s post hoc test was used to determine the influence of solvent used for hydrogel preparation on MRI signal, determination of the immune response of MyD88 and MD2 cells, and analysis of the impact of the hydrogels on GRPs viability as well as the dependence of the MRI signal on the type of hydrogel. Student’s t-test was used to compare the width of intrathecal space before and after hydrogel transplantation. All numerical data are presented as mean with standard deviation (SD), and differences were considered as statistically significant at the 95% confidence level (*p* < 0.05).

## 3. Results

### 3.1. Effect of Solvent on MRI Signal of Hydrogels

**Figure 1:**
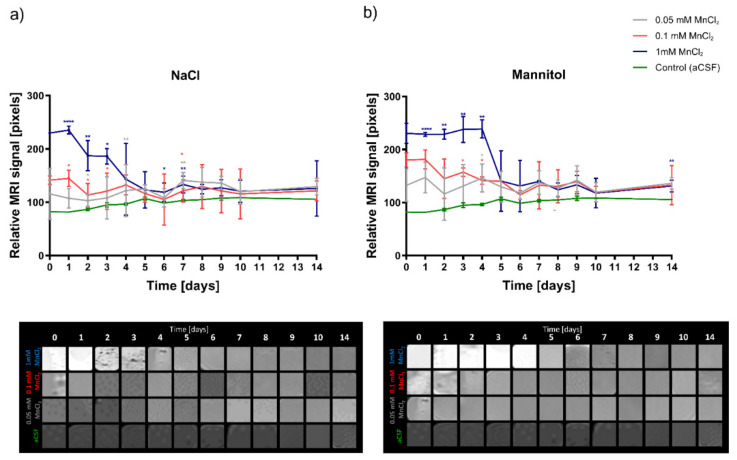
Optimization of the MRI signal of hydrogels in different diluents. (**a**) LVM hydrogels dissolved in 0.9% NaCl, (**b**) LVM hydrogels dissolved in mannitol. Data are expressed as mean with standard deviation (SD) and differences were considered as statistically significant at the 95% confidence level (* *p* < 0.05, ** *p* < 0.01, **** *p* < 0.0001).

Based on the T1 weighted MRI scans, we evaluated the signal intensity of the hydrogels, depending on the solvent used for their preparation. Hydrogels dissolved in mannitol produced a more stable signal, with less manganese-specific signal loss over time compared to hydrogels dissolved in NaCl (Figure 1). A stable signal was observed in the mannitol-based hydrogels up to day 4, while the NaCl-dissolved hydrogels lost signal strength from day two after starting the experiment. Depending on the concentration used, different degrees of T1 signal amplification were observed. The best enhancement was noted with the addition of 1 mM MnCl_2_ for both mannitol and saline hydrogels (Figure 1).

### 3.2. In Vitro Characterization of the Immune Response of the Hydrogels and Their Influence on the msGRPs Viability

**Figure 2:**
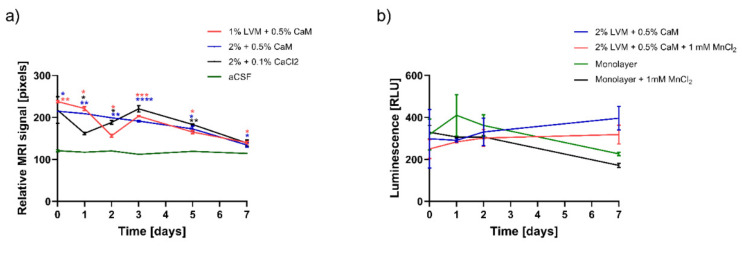
Hydrogels immune response and their influence on the GRP cells viability. (**a**) Response of the THP1-XBlue™-MD2-CD14 and THP1-Xblue™ -defMyD cells on the hydrogel components. (**b**) Viability of the GRPs embedded in the LVM hydrogels. Data are expressed as mean with standard deviation (SD) and differences were considered as statistically significant at the 95% confidence level (* *p* < 0.05, ** *p* < 0.01, *** *p* < 0.001 **** *p* < 0.0001).

To assess whether our hydrogel composition elicits an immune response, we used the MyD88 and MD2 cell lines. As indicated in Figure 2, there were no statistically significant differences in the response of both cell lines to LVM sodium alginate and calcium alginate beads of different sizes (Figure 2a). The effectiveness and reliability of the tests were confirmed by a significant increase in absorbance in samples containing positive controls-TriDap for THP1-XBlue™–defMyD88 and LPS for THP1-Xblue™-MD2-CD14 cell line. We used a luciferase-based assessment of overall cellular metabolism regarding the viability of cells inside the hydrogel matrix. As shown in Figure 2b, we compared the cells embedded in the hydrogel composition to the monolayer. Although the mean viability of the hydrogel-embedded cells (measured as the relative luminescence of luciferase-positive cells) was lower initially, this value increased after one week, exceeding the viability of the adherent cells (Figure 2b).

### 3.3. In Vitro Characterization of the Dependence of the MRI Signal on the Type of Hydrogel

**Figure 3:**
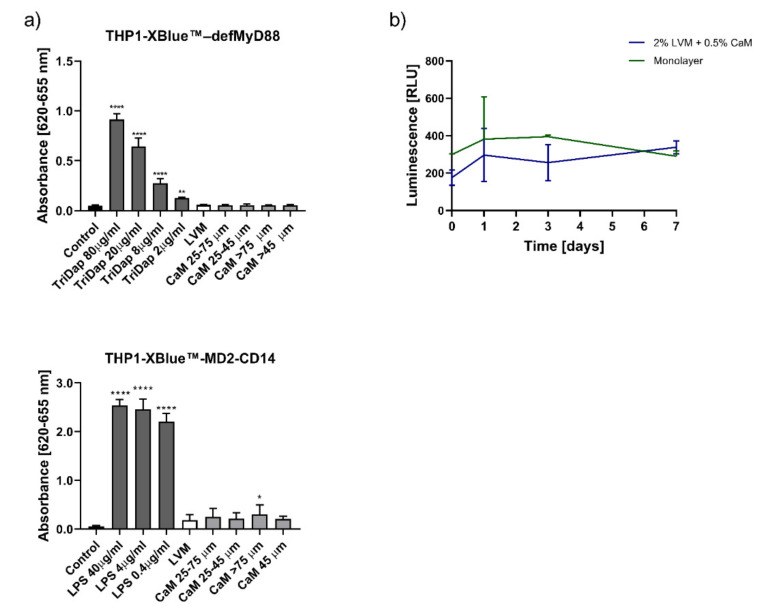
Optimization of the injectability of the hydrogels. (**a**) Influence of the percentage of the alginate and cross-linker on the MRI signal stability, (**b**) influence of the addition of the MnCl2 on the GRPs viability. (* *p* < 0.05, ** *p* < 0.01, **** *p* < 0.0001).

We assessed the loss of manganese-specific MRI signal over time for different hydrogel compositions (Figure 3a). It was shown that 2% LVM cross-linked with 0.5% CaM, injected through an epidural catheter, provided the most stable manganese release dynamics compared to 1.5% LVM with 0.5% CaM as well as to 2% LVM with 0.1% CaCl_2_, illustrated by the signal intensity profile. The detection of the signal of this hydrogel was possible 4–5 days well above the control signal. The effect of manganese supplementation on msGRPs viability was investigated using luciferase assay (Figure 3b). Our research showed a reduction in the viability of msGRPs both in adherent culture and those embedded in a hydrogel with Mn^2+^ addition. The manganese-free hydrogel composition provided a supportive microenvironment for cell growth. Higher viability of cells embedded in the hydrogel was demonstrated compared to the adherent culture control, both with and without Mn^2+^ (Figure 3b).

### 3.4. Intrathecal Injection of Mn^2+^ Hydrogels in Swine under Real-Time MRI

**Figure 4:**
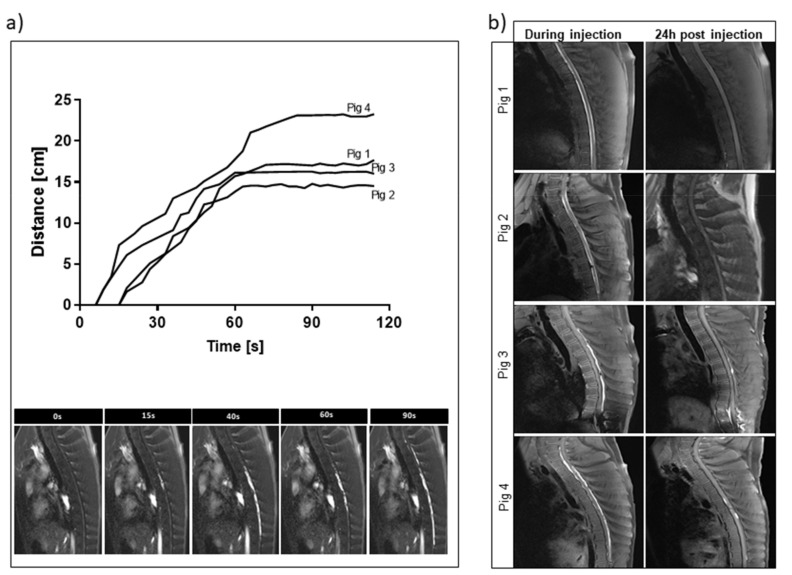
MRI-guided intrathecal transplantation of LVM/Mn2+ hydrogels. (**a**) Real-time analysis of the distance spreading of the hydrogel. (**b**) Comparison of the T1weighted MRI scans during and 24 h after injection.

To test the utility of our hydrogel composition in vivo, we performed an intrathecal injection in naïve juvenile pigs. All the injection procedures were performed under MRI guidance, which provided instant information about the hydrogel biodistribution. During the procedure, a hydrogel was visible, spreading in the intrathecal space in the rostral-caudal direction (Figure 4). The distance of the spinal cord tissue covered with the hydrogel was between 14.50 and 23.28 cm (Figure 4a). The T1 signal was excellent at the time of injection of the hydrogel, and it decreased quite rapidly to the background level throughout 24 h (Figure 4b).

**Figure 5:**
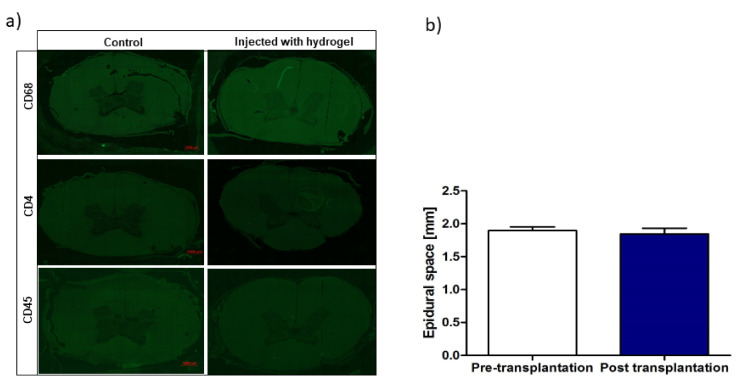
Safety of the LVM hydrogel intrathecal transplantation. (**a**) Immunofluorescence stainings of CD4, CD68, CD45 of the porcine spinal cords in the area when the hydrogel was injected. (**b**) Evaluation of intrathecal space dimensions visible on the MRI before versus post-transplantation.

In order to test whether the hydrogel graft induces any cellular response in the spinal cord tissue, we performed immunofluorescence staining for inflammatory markers. There was no evidence of cellular infiltration at the site of hydrogel injection one day after transplantation compared to the control (Figure 5A). Moreover, the procedure’s safety was confirmed by measuring the intrathecal space based on MRI images before and after transplantation (Figure 5B). There was no difference between the width of the space before and after transplantation, which is consistent with our earlier observation [11] that intrathecal injection of the hydrogel results in a long and thin deposit in the intrathecal space that does not affect the circulation of CSF.

## 4. Discussion

Divalent manganese ions (Mn^2+^) act similarly to other paramagnetic ions such as gadolinium or copper (Gd^3+^, Cu^2+^), shortening the T1 relaxation and increasing signal intensity. Work on the use of Mn^2+^ in MRI has been going on for decades and includes mainly imaging of the central nervous system [18,19], cardiovascular system [20], and cancer [21,22]. Routinely, manganese was used as a contrast in the form of manganese chloride, dissolved in water or NaCl [22,23] or as an FDA-approved contrast agent for human use—Mangafodipir [24] was usually administered intravenously, intraperitoneally, or intraparenchymally. In the current work, we proposed exploiting the unique properties of manganese as a contrast enhancer in T1 MRI and its cross-linking capacity in conjunction with alginate biomaterials used as a matrix for transplanted cells. We developed a sodium alginate-based hydrogel with physicochemical properties that allow injectability and are suitable for injecting therapeutic agents through an intrathecal catheter. T1 enhancing properties of Mn^2+^ enable visualization of the labeled hydrogel by MRI. The optimized hydrogel is immune-neutral and safe for both the embedded therapeutic cells and the recipient’s spinal cord tissue. We showed that the proposed procedure of delivering the hydrogel via the intrathecal route is feasible and safe in the highly clinically relevant setting of a large animal.

Since it is known that the various diluents affect the structure and strength of hydrogels, especially alginates [25], we studied the effect of dissolving hydrogels in NaCl compared to mannitol. Mannitol-based hydrogels showed a more consistent MRI signal, so this composition was used for in vivo experiments. Notably, mannitol has been used as a diluent to create injectable alginate-based hydrogels in the diabetes mellitus model [26]. Since the degree of signal amplification by manganese ions in T1 is closely related to its concentration, and too low and too high concentration result in suboptimal contrast, we tested three different concentrations, with 1 mM showing the greatest enhancement in the T1 sequence [27]. These results are consistent with the data obtained by Nofiele et al., who revealed that breast cancer cells had the highest degree of signal amplification in the T1 in vitro using a concentration of 1 mM of MnCl_2_. As with the research of Espona-Noguera et al. [26] and in our previous studies [11,28], injectability was also a key feature in optimizing our hydrogels. Thanks to this function, the transplanted hydrogel, unlike more solid scaffolds, can be administered non-invasively without the need for risky surgical procedures. As we report here, the hydrogel with the best injectability, but also with the most stable signal in the MRI, was based on 2% LVM, cross-linked with Mn^2+^ and 0.5% CaM. This is likely to be related to the optimal rheology of this gel, shown by Larsen et al. [29].

Apart from the useful feature of MRI visibility, our optimized biomaterial had to fulfill a supportive extracellular matrix for the embedded cells. It has been shown that pathogen-associated molecular patterns (PAMPs) can be found as impurities associated with some alginate products, which in turn results in a strong immune response [30]. For this reason, we used immunoreactivity assay based on cells expressing TLRs that recognize PAMPs. Studies by Paredes-Juarez et al. [31] on alginate hydrogels showed that they exhibit TLR-related immunoreactivity. Contrary to their research, our results did not show a statistically significant response of the two cell lines used to any hydrogel component compared to the control. This discrepancy may be related to the high purity of our alginate biomaterials. Interestingly, msGRPs cultured in alginates in the 3D system for one week showed better viability than the monolayer culture of msGRPs, indicating the positive effect of LVM/Mn^2+^ on cell viability and proliferation. In addition to the hydrogel itself, manganese can also affect viability and proliferation. Depending on the type of cells, the permissible manganese concentration is different. For lung epithelial cells, the viability reducing manganese dose was 20 mM [32], while for human osteoblasts, the concentration of 1 mM caused a significant decrease in proliferation [33]. Our research showed that the addition of manganese in a concentration of 1 mM, both when tested in adherent culture and hydrogel, slightly decreased the viability of msGRPS, but without statistical significance. Therefore, the safety of the optimized biomaterial was demonstrated, both in terms of immunology and cell support, so that we could proceed with hydrogel transplantation in an animal model.

As demonstrated in various disease models, the embedding of stem cells in injectable hydrogels significantly increased their viability and retention after transplantation [34,35,36]. The use of HA hydrogels supported viability of human neural progenitor cells (ReNcells) and msGRPs after intracerebral transplantation in mice [37]. Similarly, it was shown that the protection of msGRP cells in HA hydrogel during intracerebral administration in BALB/c mice increased their survival more than 4-fold compared to naked cells [38]. In case of hydrogel-embedded cells imaging, the utility is of upmost importance. In case of modeling the pancreatic islet-like constructs, a non-invasive MRI is proved to be useful for monitoring the assessment of their subdermal injection [39]. Additionally, in case of assessing the therapeutic efficacy and graft dynamics, hydrogel–cells composite is imaged through MRI in mouse stroke model [40]. Our data indicate that Mn^2+^-labeled hydrogel also supports the viability of embedded GRPs. Moreover, our research has shown that intrathecal injection of the hydrogel results in its spreading over quite an extensive distance ranging 23 cm along the spinal cord with its good local retention. It gives great opportunity to cover a large area of affected spinal cord tissue with cells embedded in the hydrogel, thus the potential therapeutic effects.

Thanks to the possibility of imaging our hydrogel due to Mn^2+^ signal in MRI facilitating visualization of the hydrogel deposit in real-time, it will allow tuning neurointerventions with an example of adjusting the volume of injected tissue composites or changing the position of the catheter. At the same time, we showed that hydrogel transplantation is safe, does not cause an inflammatory reaction from the spinal cord tissue, and does not seem to alter the CSF circulation. The presented innovative use of Mn^2+^ as a contrast agent and a cross-linker may be an excellent solution for the transplantation of both cells and drugs in preclinical models and eventually also in patients.

## 5. Conclusions

Our study showed that it is possible to introduce manganese (II) ions into sodium alginate solution to obtain biomaterial with MRI contrast properties. We conducted a series of in vitro experiments to assess the safety of the hydrogel in terms of immunogenicity and its effect on the cells suspended in it. Our research confirmed that the hydrogel does not induce an immune response and that it has a neutral effect on the viability of stem cells. In view of the positive effects obtained in in vitro studies, we tested the safety of the hydrogel administration procedure by intrathecal transplantation into CSF. Our in vivo study showed that it is possible to perform a precise intrathecal injection of alginates supplemented with manganese under MRI guidance. Our approach made it possible to conduct imaging and short-term non-invasive monitoring of the MRI transplant. The presence of the hydrogel did not induce leukocyte migration to the area of the spinal cord tissue, indicating the biocompatibility of the hydrogel composition. Moreover, the rapid release of manganese (II) ions from the hydrogel matrix in vivo suggests the suitability of the hydrogel composition for a short term evaluation with a low risk of manganese toxicity. In case long-term tracking is desirable this composition is not suitable which we report as a limitation.

## Data Availability

The data presented in this study are available on request from the corresponding author.
